# Increased Mortality Exposure within the Family Rather than Individual Mortality Experiences Triggers Faster Life-History Strategies in Historic Human Populations

**DOI:** 10.1371/journal.pone.0083633

**Published:** 2014-01-08

**Authors:** Charlotte Störmer, Virpi Lummaa

**Affiliations:** 1 Institut für Philosophie, Justus-Liebig Universität Gieβen, Gieβen, Germany; 2 Department of Animal and Plant Sciences, University of Sheffield, Sheffield, United Kingdom; 3 Wissenschaftskolleg zu Berlin, Berlin, Germany; Nathan Kline Institute and New York University School of Medicine, United States of America

## Abstract

Life History Theory predicts that extrinsic mortality risk is one of the most important factors shaping (human) life histories. Evidence from contemporary populations suggests that individuals confronted with high mortality environments show characteristic traits of fast life-history strategies: they marry and reproduce earlier, have shorter birth intervals and invest less in their offspring. However, little is known of the impact of mortality experiences on the speed of life histories in historical human populations with generally higher mortality risk, and on male life histories in particular. Furthermore, it remains unknown whether individual-level mortality experiences within the family have a greater effect on life-history decisions or family membership explains life-history variation.

In a comparative approach using event history analyses, we study the impact of family versus individual-level effects of mortality exposure on two central life-history parameters, ages at first marriage and first birth, in three historical human populations (Germany, Finland, Canada). Mortality experience is measured as the confrontation with sibling deaths within the natal family up to an individual's age of 15.

Results show that the speed of life histories is not adjusted according to individual-level mortality experiences but is due to family-level effects. The general finding of lower ages at marriage/reproduction after exposure to higher mortality in the family holds for both females and males. This study provides evidence for the importance of the family environment for reproductive timing while individual-level mortality experiences seem to play only a minor role in reproductive life history decisions in humans.

## Introduction

Life-histories (LH) are characterized by the timing of life events like sexual maturation, reproduction and death. Life histories differ both between (see [1] for differences in mammalian LH) and within species. These differences might be apparent either at the (sub-) population level [2], [3], [4] or at the family/individual level [5], [6], [7]. However, to our knowledge, the relative importance of the effects of family environment (conditions shared by all family members) and the impact of individual experiences (e.g. individual differences within the family in timing and occurrence of experiences due to birth order) have not explicitly been addressed yet.

One of the most important environmental cues influencing the variation in the speed of life histories between populations and individuals is the extrinsic mortality risk (e.g. [8],[9],[10]). However, whereas the impact of environmental factors such as food availability (e.g. [11]) or stressful events early in life (e.g. [12]) on human life-histories is well documented, more attention should be paid to high-mortality regimes that have potentially links to faster ontogeny and may contribute to important variation in modern human growth and development [10]. A recent approach characterized by the “dying young and living fast”-concept (based on Nettle [4]) addresses LH differences at the (sub-) population level in relation to mortality/morbidity risk. Such studies have shown that fast LH strategies, such as early menarche and reproduction (high incidence of teenage pregnancies) and low parental investment in offspring dominates among females living in modern western environments characterized by high morbidity/mortality and hence reduced mean life expectancy (e.g. [13],[14],[15],[4],[16]). Cues of risky environments such as crime and insecure social conditions are also linked to faster LH strategies (e.g. [17]). The underlying algorithm for faster life histories is: Mature fast and reproduce early (and often) to increase the chances of leaving at least some offspring in risky/insecure environments. Little is known, however, about the impact of mortality risk on male reproductive behaviour in humans. This is probably due to the fact that females show much clearer indicators of their reproductive development and timing (age at menarche, birth, menopause) than males, leading to studies on males focussing on effects of early stressors on surrogate outcomes such as violence, aggression or age at first sex (e.g. [18],[19],[20]). The only study we know of addressing male reproductive timing in relation to early mortality risk is by Störmer [21] on a historical population of the German coastal region Krummhörn, where males confronted with high levels of extrinsic mortality (exposed to smallpox epidemic prenatally or up to age 2) reproduced earlier and had a reduced proportion of surviving offspring than non-exposed males.

Above these specifically health and life-expectancy related aspects, there is also evidence that early stressful (family) environments can trigger faster life-history strategies in humans (for females see e.g. [5],[22],[23],[24]; see [25] for a study on male sexual maturation and reproductive timing). Belsky and colleagues [5] for example, point out the importance of stable family constellations and the timing of stressful events. They argue that due to the plasticity of developmental processes early in life, environmental cues can shape the development and hence can have an impact on human life histories. Divorced parents, for example, with the resulting stressful family environment can be an indicator for low parental investment. Several studies have showed that early exposure to such stressful family environments leads to early menarche and early first reproduction among daughters (e.g. [26] and [24]). Furthermore, early stress is related with instable pair-bonding in adulthood with short-term partner relationships favoured ([23],[24]). As the early environment is in a large part characterized by parental behaviour, behavioural adaptations of children will depend on these cues. In (socially) insecure and stressful environments, an adjustment to these conditions resulting in fast life-history traits such as early menarche and reproduction could be evolutionary advantageous if the early environment is a good predictor of future living conditions, too.

In summary, there is plenty of evidence that stressful early environments, characterized by high levels of extrinsic mortality, morbidity and family instability are related with fast life-history strategies in humans, especially in females. However, three gaps in our knowledge remain. First, most previous studies have focused on contemporary populations characterized by relatively low mortality rates even among the “high mortality” groups as compared to historical, pre-industrial human populations or current “traditional” populations where childhood mortality levels can reach ∼40% (e.g. [27]). Some of such studies have found that mortality exposure shows a quadratic relationship with the timing of first birth, with age at first birth decreasing as mortality exposure initially increased, but at the highest measured mortality levels, increasing age at first birth [28]. This leaves it open how mortality levels far higher than those studied so far influence the speed of human life histories. Second, in most previous studies there is no clear distinction between experiences and consequences at the family and the individual level. Individual level experiences are those experiences that are not shared by all family members. For example, depending on their birth rank, siblings will not be exposed to the same environmental cues. Moreover, the timing of the confrontation with a specific event will not be the same for all family members and experiences might therefore have different effects on different family members. Exposure to mortality in early years, for example, has been found to have a stronger influence on long-term fertility behaviour than exposure in later life ([28],[29]). Conclusions about whether the impact of individual experiences or the family context (shared characteristics like e.g. the family environment, family size, or genetic predispositions) is more important for differences in LH strategies are therefore hard to draw from current studies. Finally, given the notable differences in male and female life histories and age-specific fitness gains in humans (e.g. [30]), more studies are needed on the effects of mortality risk on life-history patterns in males to gain a better understanding of the similarities and differences in the effects between the sexes.

To fill these empirical and theoretical gaps, this study uses individual-based longitudinal data to investigate the impact of mortality exposure during infancy and childhood on reproductive behaviour of females and males in three historical (sub) populations characterized by relatively high mortality rates as compared to previously studied populations (historical pedigree data (17^th^ to 19^th^ century) of the Krummhörn region (Germany), five Finnish parishes and the Québec Region (Canada)). We focus on two different levels of mortality exposure:

1. Family level: Does reproductive timing differ among families according to mortality exposure within the family? More precisely, does the exposure to sibling deaths within the natal family up to age 15 have an impact on reproductive timing of females and males in historical societies? Humans grow up in a family context and the family environment is therefore the most important point of reference for environmental cues, especially during the first years of life. The family environment can be a good predictor of future environmental conditions which an individual will have to face. Mortality exposure within the natal family as one specific shared environmental cue early in life might therefore influence the development of the life-history strategy in relation to mortality risk and life expectancy.

2. Individual level: Does individual mortality experience within the natal family result in individual life-history adjustments or does family membership explain most of the variation in life-history strategies? In other words, does reproductive timing of siblings differ according to the confrontation with sibling deaths within the same family which differ in number and timing? For example, firstborns cannot experience sibling death very early in life (except of twin births) as they do not have elder siblings. Hence, siblings will be exposed to different numbers of sibling deaths at different stages in development and even within families mortality experiences will vary between individuals. Environmental cues influencing life-history strategies should affect an individual as directly as possible to enable increasing reproductive success in a given environment. However, such individual level life-history adaptations with respect to mortality risk have not been studied yet. To our knowledge, this is the first study which addresses whether family membership (genetic predisposition/shared environment) or individual experiences trigger faster life histories in humans.

## Material

This study is based on family reconstitution data from church records and tax rolls of three different historic populations: the Krummhörn (18^th^ and 19^th^ century), five Finnish parishes (18^th^ and 19^th^ century) and the Québec region in Canada (17^th^ and 18^th^ century). The historical Krummhörn was a small coastal region in north-western Germany (for a detailed introduction to the dataset see [31]). The Krummhörn comprised 32 parishes. To date, 27 of these parishes have been reconstituted and compiled into a database. Life data on approximately 64,000 individuals is available. Due to the geographical position, expansion possibilities for the Krummhörn population were limited [32] and the population faced a displacement competition. This was reflected by a low mean number of children (4) in complete families, as compared to other historical populations. Above that, the Krummhörn population was characterized by relatively low infant mortality (∼12%) and did not suffer from famines during the study period (18^th^–19^th^ century).

The Finnish dataset ([33],[34]) is based on pedigree data from five Finnish parishes. Two of these are mainland parishes while the other three are located in the archipelago region. The version of the dataset analysed here comprises data on life histories of approximately 54,000 individuals. People made their living by agriculture, sometimes supplemented by fishing. Living conditions can be described as natural fertility and mortality conditions: Women gave birth to five children on average and about 40% of all born individuals died before reaching adulthood [11]. Famines were relatively common due to frequent crop failures [35].

Data on the Québec region in Canada has been collected by the ‘Programme de recherché en démographie historique’ (PRDH) at the University of Montréal (for more information see www.genealogy.umontreal.ca). This data dates back to the first French settlers in the St. Lawrence valley during the 17^th^ century and contains life data of about 307,000 individuals.

Geographical and socio-ecological living conditions of the Québec population differ from other historical populations such as the Krummhörn (for a comparison of these study populations see also [36]). Land was unlimited for settlers. Many of them founded new farms and hence the population grew and faced an expansion competition. Fertility in this population was high: women gave birth to 10–11 children on average. At the same time, infant and child mortality were high as well: 22% of the children died during infancy and only ∼60% survived until they reached age 20 [37]. Mortality risk, however, differed between urban and rural regions [38] and was higher in the urban part of the population.

### Data selection

To analyze the impact of early mortality exposure on human reproductive timing, we selected individuals from each of the three datasets to be included in the present study according to the following criteria. Individuals had to be born in the first marriage of both their father and mother (only full siblings are considered in the analysis), and their date of birth, marriage and/or birth of the first offspring were known. To analyze the mortality exposure within the natal family, date of death had to be known also for all siblings. As the social upper class of large-scale farmers in the Krummhörn is small and known to differ in reproductive behaviour from the rest of the population [39], farmers with a landownership of more than 75 grasen (∼28 ha; see [40]) were excluded from the sample. Each database used in this study has its own record related limitations. Therefore, the databases do not necessarily cover the same time periods and different time windows for data selection had to be defined. All individuals included in the present study from the Krummhörn and the Finnish datasets were born between 01.01.1750 and 31.12.1829. For the Québec population, data were selected by the parental date of marriage (01.01.1670–31.12.1720). After data selection, the sample sizes are as follows for age at first marriage: Krummhörn: 967 men and 1,064 women; Finland: 1,448 men and 1,542 women; Québec: 15,433 men and 15,350 women. As children are not reported for all married individuals (due to e.g. infertile marriages, emigration or death), sample sizes for age at first birth are slightly lower. For a detailed summary of the datasets see table 1.

**Table 1:** Descriptives for the study populations (Krummhörn, Finland and Québec) including sample sizes, mean ages at marriage and first birth as well as proportions of individuals confronted with mortality separated by sex.

Descriptives		Sex	Krummhörn	Finland	Québec
**No. of individuals (total)**	Age first marriage	Male	N = 967	N = 1,448	N = 15,433
		Female	N = 1,064	N = 1,542	N = 15,350
					
	Age first birth	Male	N = 875	N = 1,305	N = 14,124
		Female	N = 940	N = 1,392	N = 14,017
					
**No. of individuals with mortality exposure**	Age first marriage	Male	N = 327 (∼34%)	N = 575 (∼40%)	N = 6928 (∼45%)
		Female	N = 310 (∼30%)	N = 558 (∼36%)	N = 8317 (∼54%)
					
	Age first birth	Male	N = 310 (∼35%)	N = 526 (∼40%)	N = 6389 (∼45%)
		Female	N = 296 (∼31%)	N = 535 (∼38%)	N = 7548 (∼54%)
					
**Mean age at first marriage [years]**	No mortality exposure	Male	29.35+/− 5.76	26.66+/− 5.48	28.32+/− 6.31
		Female	27.21+/− 5.39	25.40+/− 5.58	23.00+/− 5.56
					
	Mortality exposure	Male	28.80+/− 5.05	26.48+/− 5.00	26.92+/− 5.20
		Female	26.84+/− 5.61	26.46+/− 5.91	22.89+/− 5.34
					
**Mean age at first birth [years]**	No mortality exposure	Male	29.79+/− 5.84	29.34+/− 6.78	29.17+/− 6.15
		Female	27.18+/− 4.82	27.01+/− 5.51	23.74+/− 4.77
					
	Mortality exposure	Male	29.25+/− 5.28	27.93+/− 6.16	27.97+/− 5.17
		Female	26.76+/− 4.59	26.61+/− 5.00	23.64+/− 4.77

Note: Mortality exposure refers to the exposure to sibling deaths before the focal individual reaches age 15.

## Methods

We use event history analysis to analyze the impact of mortality exposure within the family on the speed of human life histories (STATA 8.0 special edition; for an introduction to survival and event history analyzes, see e.g. [41],[42] or [43]). Event history analysis (EHA) is based on a semi-parametric Cox regression. Survival analyses based on Cox regression modelling is a standard procedure in time to event analyses (e.g. [44],[45]). As it is a semi-parametric method, no specific assumptions about the distribution of the data are necessary. The benefit of such survival analyses is that they incorporate information on the timing of events into the models. This enables more detailed modelling of the impact of specific events on later events (e.g. the confrontation with deaths within the family on reproductive timing) as not only the event (death) itself but also the timing of the event is included.

Standard EHA models postulate the proportionality of hazards which implicates that the covariates have multiplicative effect on the hazard function [43]. If this proportionality assumption is not met because the effect of some covariates on the hazard function changes over time, EHA models can be adjusted by including specifications accounting for covariates showing an interaction with time. These models will be referred to as iwt-models (interaction with time).

To test whether individual experiences or family membership explains differences in life histories, stratified event history analyses have been used. Stratifying on families (full siblings) means that for each family (sibship) a separate baseline is allocated (but not estimated). Such a model compares individuals from the same family and therefore controls for shared family factors (for a more detailed description see [45]). Stratified models are very similar to random effects models but they leave any distribution of differences between strata unspecified and make no assumption about the relative form of any given strata [46].

The dependent variables in all statistical procedures are age at first marriage and age at first birth. These two events are key life-history traits (see e.g. [15]) and characterize life-history strategies because they mark the beginning of the reproductive period. All models use the death of full siblings up to the 15^th^ birthday of the focal individual as a measure of within-family mortality experience as the variable of interest. This variable represents individual mortality exposure during infancy and childhood, hence during those developmental periods which are most likely sensitive to environmental cues.

In a first step we use common EHA with time-specifications of the covariates if the proportionality assumption is not met. Beside the mortality variable all models include the main terms of individual sex and his/her total family size (number of siblings). Additionally, we tested for interaction terms that investigate possible sex-related differences in the impact of early mortality exposure (sex*mortality) and for possible effects of the family size (number of siblings) up to the age of 15 (family size*mortality).

In addition to these focal parameters, all models control for various covariates. As there is evidence that the loss of parents is an important mortality cue affecting survivability and hence human life-histories (e.g. [47],[48] and [49] for historical populations) we control for these effects by including the individual's age at father's and mother's death in all models. The child's age at loss of the parent is divided into the following categories: <1 year (infancy), 1–4 years (early childhood), 5–14 years (childhood), >15 years (adulthood). The reference for both sexes is parental loss during adulthood (age group >15 years).

Birth rank (included in the models as a continuous variable) controls for possible effects of birth order on reproductive decisions. In addition to the above mentioned covariates, the models also control for the birth cohort (coded in decades) to adjust for general time trends in mortality and reproduction. As all models are conducted separately for the three different populations we also control for some population-specific factors: models on the Finnish dataset control for the birth parish of the individual (five levels) and models on the Québec dataset control for urban birth of the individual to take into account spatial variation in mortality and reproduction.

In a second step, to study the impact of individual level mortality experiences on reproductive timing, we use stratified event history models. Stratification by family ID (same ID for all offspring of one family) controls for effects due to shared family environment and/or genes, and hence shows whether effects of mortality experiences are due to family membership or an adjustment to individual experiences within the family. The stratified models compare reproductive timing of siblings according to individual mortality exposure. The stratified event history models use the same response variables and parameters as described above for the traditional EHA models.

## Results

Table 1 shows descriptive statistics for the three study populations. The populations differ in their mean age at first marriage and first birth with Québec women being youngest and Krummhörn women being oldest at these reproductive events: While women in Québec marry at an average age of 23 (+/− 5.56 years) women in the Krummhörn marry in their late twenties (27.21+/−5.39 years). There is no such clear trend in males. The proportion of individuals confronted with mortality events in the family also differs between the different populations. While only ∼30–35% of the individuals of the Krummhörn sample are confronted with sibling deaths, more than 45% of the Québec individuals are confronted with sibling deaths.

### Event history models

Our results show that both sexes tend to marry and reproduce earlier when confronted with mortality in their natal family. All models incorporate time-dependency of covariates as the proportionality assumption was not met. Event history analyses for the Krummhörn population (table 2) show that mortality exposure significantly reduces the age at first marriage (HR = 2.72 *) in the iwt-model for both sexes (interaction between sex and mortality exposure is non-significant). Above that, the interaction between family size and mortality significantly affects age at first marriage in the iwt-model with mortality exposure significantly decreasing age at first marriage in small families (with less than the mean number (5) of siblings, separate models not shown). Both effects show an interaction with time (iwt). In the same way mortality exposure (HR = 1.20 *) and the interaction between family size and mortality affect age at first birth in the Krummhörn.

**Table 2:** Results for the Event History Analysis on age at first marriage and age at first birth in relation to early mortality exposure for the Krummhörn, the Finnish, and the Québec population.

Krummhörn				
	Age at first marriage		Age at first birth	
Model	iwt	strata	iwt	strata
N subjects	2,019	2,019	1,806	1,806
N observations	2,961	2,961	2,716	2,716
Mortality experience	2.72 * (iwt)	.32	1.20 *	1.21
Sex	.14 *** (iwt)	.06 *** (iwt)	.20 *** (iwt)	.10 *** (iwt)
Sex*mortality	1.00	.90	1.04	1.09
Family size*mortality	.86 * (iwt)	1.12	.97 *	.97
Birthcohort	.77 *** (iwt)	.45	.86 * (iwt)	1.13
Family size	.97	1.46	.97 *	.94
Birthrank	1.08	1.17	1.07 **	.97
Model parameters				
LR chi^2^	160.50	90.55	154.08	88.58
Chi^2^	0.0000	0.0000	0.0000	0.0000
Log likelihood	−13255.944	−1072.2759	−11657.559	−873.46121
**Finland**				
N subjects	2,528	2,528	2,227	2,227
N observations	4,423	4,423	3,985	3,985
Mortality experience	1.00	.85	1.03	.88
Sex	.36 *** (iwt)	.14 *** (iwt)	.71 ***	.67 ***
Sex*mortality	1.13 ***	1.16 *	1.07+	1.11
Family size*mortality	.99	1.01	.99	1.00
Birthcohort	1.00	1.01	1.01	1.04
Family size	.84 *** (iwt)	.60	1.01	.92
Birthrank	1.02	1.02	1.00	.98
**Model parameters**				
LR chi^2^	107.00	68.39	77.19	40.86
Chi^2^	0.0000	0.0000	0.0000	0.0237
Log likelihood	−17231.252	−1391.1835	−15069.77	−1256.9297
Québec				
N subjects	24,831	24,831	22,778	22,778
N observations	52,151	52,151	47,481	47,481
Mortality experience	1.04 **	1.04	1.13 *** (iwt)	.95
Sex	.02 *** (iwt)	.00 *** (iwt)	.02 *** (iwt)	.00 *** (iwt)
Sex*mortality	1.02	1.02	1.02 +	1.01
Family size*mortality	1.00	1.00	1.00	1.00
Birthcohort	.90 *** (iwt)	.78 * (iwt)	.85 *** (iwt)	.94
Family size	.96 *** (iwt)	.96	.94 *** (iwt)	.90 * (iwt)
Birthrank	1.02 ***	.99	1.02 ***	.99
Urban birth	1.23 ** (iwt)	1.05	1.26 ** (iwt)	1.34
**Model parameters**				
LR chi^2^	5084.66	5031.92	5002.05	4613.59
Chi^2^	0.0000	0.0000	0.0000	0.0000
Log likelihood	−223923.57	−22807.987	−203275.8	−19849.948

Results are shown for both the iwt-models (controlling for covariates showing an interaction with time if the proportionality assumption is not met) and the stratified model (stratification by family ID to control for family membership). The effects of the included covariates are reported as hazard ratios (HR). Sex is coded as 0 =  female and 1 =  male. For complete results of the models including all covariates see the supporting information (tables S1, S2 and S3).

iwt  =  controlling for covariates showing an interaction with time.

Significance: *** p<0.001; ** p<0.01; * p<0.05; +p<0.1.

In the Finnish population, mortality exposure had different effects on the reproductive timing in males and females, as evidenced by a significant interaction term between sex and mortality exposure (HR = 1.13 ***). Mortality exposure increased age at marriage in females and decreased it in males (both effects are marginally significant, separate models not shown). Similarly, mortality exposure increased age at first reproduction among females and decreased it in males, but this interaction was only marginally significant (table 2).

In the Québec population mortality exposure early in life significantly reduced both age at first marriage (HR = 1.04 **) and age at first birth (HR = 1.13 ***) in the iwt-models in both sexes (table 2). The decreasing effect of mortality exposure on age at first birth shows an interaction with time, it is highest during infancy and childhood. Above that, age at first marriage and first birth are higher in bigger families (HR = .96 *** and HR = .94 ***, respectively).

### Stratified event history models (individual level effects)

The stratified event history models control for family membership. Only those effects of mortality exposure showing up in these models are attributable to individual mortality experiences, not only to the impact of family membership. Stratification for the Krummhörn as well as for Finland and for Québec shows no significant impact of individual mortality experiences within the natal family on reproductive timing (see table 2). This holds for both reproductive parameters (age at first marriage and age at first birth) and both sexes, confirming that the impact of mortality exposure on reproductive timing detailed above is attributable to family-level experience and membership, rather than individual experiences.

### Control variables

All event history models control for various covariates (results are shown in table 2 and in the supporting information in tables S1, S2 and S3). The number of siblings up to the age of 15 increases the age at marriage and the age at first reproduction, respectively, in all study populations. At the same time, the individual's birth rank affects age at reproduction in the Krummhörn and the Québec population: the higher the birth rank, the lower the age at first reproduction.

Controlling for effects of the parents shows that the individual's age at death of father or mother decreases age at reproduction in some of the age groups in the Krummhörn and the Québec population. In general, parental death has a decreasing effect on the reproductive age while early loss of the mother is associated with earlier marriage in the Krummhörn (tables S1) and parental loss during the reproductive ages decreases age at marriage and first birth in Québec (table S3).

Birth cohort has an increasing effect on the age at reproduction in the Krummhörn and in Québec. This effect shows an interaction with time in both populations.

Birth parish in the Finnish population does not have any effect on reproductive timing (table 2). Urban birth in the Québec population decreases age at reproduction which means that individuals born in the urban areas marry and reproduce significantly earlier than individuals born in the rural areas (table 2).

## Discussion

Although extrinsic mortality risk is predicted to modify the speed of life histories between populations and individuals (e.g. [8],[9],[10]), little is known on how the effects compare between the sexes in human populations characterized by high general mortality rate. Furthermore, most previous studies do not distinguish between mortality experiences at the family and the individual level. Our study demonstrates a link between extrinsic mortality rate and life-history strategies in three different historic human populations. As predicted by the life-history theory, life histories of both males and females with high levels of mortality exposure in childhood (experience of sibling deaths) were characterized by earlier marriage and/or earlier first birth and, hence, faster life histories. Our results stress the importance of the family environment rather than individual experiences in modifying reproductive timing and life-history decisions in humans.

The current literature has focused on environmental effects on life histories of women only, and it is thus of interest that we found no general sex differences in the impact of early life mortality experiences on reproductive timing. Hence, the effects of mortality risk on the speed of life histories does not appear to be female-specific. The question then arises why both sexes adjust their life-history strategies in relation to early mortality experiences. Male and female life histories and age-specific fitness gains differ in humans with males continuing to gain reproductive success into later age (e.g. [30]). It has been argued that male investment in reproduction is much lower than that of females and therefore risky (reproductive) strategies associated with fast life histories are on average more beneficial for males [13]. On the other hand, especially males might be sensitive to morbidity and mortality conditions as males are more vulnerable themselves (e.g. [50]). In high morbidity/mortality environments particularly males might thus have faster life histories to increase their chance of reproducing at all ([1],[51],[21]). In the light of our results, it is likely that both sexes react to cues of environmental stability and predictability such as mortality risk, although not necessarily to the same extent in all conditions.

Another novel question of this study is whether individual mortality experiences within the natal family result in life-history adjustments or whether family membership alone explains most variation in life-history strategies. In general, the results indicate that the impact of mortality exposure on male and female reproductive behaviour in the different populations is due to family membership as there were no mortality-specific effects in the stratified event history models. These results at the family level are consistent across populations and therefore seem to be independent of socio-ecological conditions and mortality regimes. However, in the Krummhörn population the effects of mortality exposure are more pronounced in smaller families indicating that the proportion of siblings dying within the family is one important parameter of extrinsic mortality risk. One possible explanation why family is the most important level of environmental conditions affecting life-history strategies is that the results might be caused by some kind of ‘family mentality’: parental behaviour which is sensitive to environmental stress will be reflected in their children. Chisholm argues that children are not able to perceive mortality risk directly [14] but instead, loss of children will most likely result in parenting behaviour which confronts the surviving offspring with an insecure environment, subsequently reflected in their life-history. The cues of the rearing environment thus have an impact on the development of an individual reproductive strategy (see also [8]), and such a family environment will be the same for all children of a given natal family. The ‘family mentality’, mediated by psychological mechanisms, will thus increase the similarity of sibling's life-history strategies, and mask individual (different) mortality experiences among siblings.

Above that, we would like to draw attention to the debate that humans are cooperative breeders (e.g. [52],[53]). If arguments will confirm this idea it will also emphasize that reproductive strategies are always part of a cooperative breeding system. As information on family members as potential partners in rearing offspring is very important in such a context, reproductive decision-making procedures are more likely to be collectively than individually oriented. Hence, environmental cues at the family level would be even more likely to be relevant for the development of specific reproductive strategies.

Besides direct environmental effects of mortality experience on life-history speed, other possible explanations for the importance of family (environment) on human life histories include genetic correlates of life-history parameters. First, siblings share genetic traits and are, hence, more similar in behavioural traits than non-related individuals. For example, heritability estimates are quite high for different aspects of fertility ([54], e.g. for age at first reproduction [55]). Therefore, sisters will be similar in timing of reproductive events. Second, it has been shown in several populations that offspring deaths tend to cluster within families and only a few families (women) are responsible for a high proportion of infant/child deaths (e.g. [56],[57]). Consequently, the risk of being exposed to sibling death early in life varies heavily between families. Both genetic relatedness and close cohabitation of relatives increase the chance of exposure to the same environmental conditions, possibly leading to family-level links between exposure to mortality within the natal family and age at the onset of reproduction. Few direct studies addressing this possibility exist, however. Studies on child survival in migrant families have found that living conditions at the new place of residence did not change child survivability considerably (e.g. [58] for developing countries; [59] for Senegal) and family membership rather than environmental conditions (measured by place of residence) explained most variation in survivability. Third, it might be argued that the effects of mortality exposure on reproductive timing are a product of socio-economic status related mortality and reproduction. However, this is unlikely as the social upper class of the Krummhörn has been excluded from the models and in Québec ‘urban birth’ controls for differences in living conditions between urban and rural regions. In Finland, the poor have the highest child mortality (e.g. [33]) but the latest age at marriage and first reproduction [60]. We therefore think that the impact of mortality exposure is robust across socio-economic status groups.

In addition to the results that family membership influences reproductive behaviour more than individual experiences and that mortality exposure affects both female and male reproduction, this study also indicates time-dependent effects of mortality exposure. Mortality exposure shows an interaction with time for age at first marriage in the Krummhörn population and for age at first birth in Québec. This means that the effect of mortality exposure on reproductive timing is strongest early in life while mortality exposure later on during childhood does not significantly affect reproduction anymore. This is in line with other studies (e.g. [5],[61],[29], [2],[25]) showing that there are “sensitive periods” in which environmental factors can have an impact on developmental/psychological processes. One possible mechanism linking early experiences with later reproduction is attachment. The attachment style develops in relation to the social environment early in life and influences social and reproductive behaviour throughout the life course. Insecure attachment, for example, results from stressful and unstable environments and is characterized by traits of fast life histories like impulsivity, short-term thinking, promiscuity, low investment in parenting and supporting behaviour and increased risk-affinity ([6],[18]). Furthermore, insecure attachment is also linked to early menarche (e.g. [26]). All of these traits are typical for fast life-history strategies. It follows that there are not only environmental constraints but also ontogenetic constraints: if the attachment style is fixed in childhood this will predetermine the reproductive strategy later in life.

However, a study by Placek & Quinlan [29] suggests that not only early mortality risk affects reproduction, but also the current risk of mortality during the reproductive period (measured at the population level) has a mediating effect on subsequent fertility. Future studies might therefore consider both early and later mortality risks (see e.g. [8] for age-dependent effects of environmental risk) to answer the question to what extend the impact of early conditions on human life history strategies can be modified later in life ([62],[35]).

To our knowledge, this is the first study addressing family and individual level effects of early life mortality exposure on reproductive strategies in humans. We show that it is the family membership with all its different aspects leading to adjustments in reproductive timing in both females and males. Therefore future studies should not only focus on female reproductive strategies in relation to extrinsic mortality and other environmental cues. To complete our understanding of environmental factors affecting development and adjustments in human reproductive strategies at the family and individual level, future studies should incorporate data from (modern as well as historical) populations experiencing a range of mortality levels, both early in life and at the beginning of the reproductive period, and a variety of reproductive parameters.

## Supporting Information

Table S1 **Results for the Event History Analysis on age at first marriage and age at first birth in relation to early mortality exposure in the Krummhörn population.** Results are shown for both the iwt (controlling for covariates showing an interaction with time) and the stratified model (stratified by family ID).
(DOCX)

Table S2 **Results for the Event History Analysis on age at first marriage and age at first birth in relation to early mortality exposure in the Finnish population.** Results are shown for both the iwt (controlling for covariates showing an interaction with time) and the stratified model (stratified by family ID).
(DOCX)

Table S3 **Results for the Event History Analysis on age at first marriage and age at first birth in relation to early mortality exposure in the population of Québec.** Results are shown for both the iwt (controlling for covariates showing an interaction with time) and the stratified model (stratified by family ID).
(DOCX)
