# Supplementary material for: Increased Mortality Exposure within the Family Rather than Individual Mortality Experiences Triggers Faster Life-History Strategies in Historic Human Populations
Source: PLoS One. 2014 Jan 8;9(1):e83633. doi: 10.1371/journal.pone.0083633 (PMC3885450; doi:10.1371/journal.pone.0083633)
Supplement: Table S1 — Results for the Event History Analysis on age at first marriage and age at first birth in relation to early mortality exposure in the Krummhörn population. Results are shown for both the iwt (controlling for covariates showing an interaction with time) and the stratified model (stratified by family ID). (DOCX) [file pone.0083633.s001.docx]

Table S1: Results for the Event History Analysis on age at first marriage and age at first birth in relation to early mortality exposure in the Krummhörn population. Results are shown for both the iwt (controlling for covariates showing an interaction with time) and the stratified model (stratified by family ID).

|  | | | | | | |
| --- | --- | --- | --- | --- | --- | --- |
|  | **Age at first marriage** | | | **Age at first birth** | | |
| Model | **normal** | **iwt** | **strata** | **normal** | **iwt** | **strata** |
| N subjects | 2,019 | 2,019 | 2,019 | 1,806 | 1,806 | 1,806 |
| N observations | 2,961 | 2,961 | 2,961 | 2,716 | 2,716 | 2,716 |
| Mortality experience | .99 | 2.72 * (iwt) | .32 | 1.23 * | 1.20 * | 1.21 |
| Sex (male) | .72 *** | .14 *** (iwt) | .06 *** (iwt) | .60 *** | .20 *** (iwt) | .10 *** (iwt) |
| Sex*mortality | 1.01 | 1.00 | .90 | 1.03 | 1.04 | 1.09 |
| Family size*mortality | 1.00 | .86 * (iwt) | 1.12 | .97 * | .97 * | .97 |
| Birthcohort | .98 | .77 *** (iwt) | .45 | .99 | .86 * (iwt) | 1.13 |
| Family size | .97 + | .97 | 1.46 | .97 * | .97 * | .94 |
| Birthrank | 1.05 * | 1.08 | 1.17 | 1.07 ** | 1.07 ** | .97 |
| Maternal age at birth |  |  |  |  |  |  |
| *14-19 years* | 2.12 | 2.16 | 1.61 | 1.36 | 1.39 | 2.39 |
| *20-24 years* | 1.05 | 1.05 | 1.14 | 1.05 | 1.06 | 1.12 |
| *25-34 years (ref)* | 1 | 1 | 1 |  | 1 | 1 |
| *35-39 years* | .89 + | .91 | .81 | .90 | .91 | .85 |
| *40-44 years* | .95 | .98 | .80 | 1.00 | 1.01 | .68 |
| *45-49 years* | 1.65 | 1.68 | 1.59 | 1.46 | 1.49 | 1.06 |
| Paternal age at birth |  |  |  |  |  |  |
| *20-24 years* | 1.09 | 1.05 | .69 + | 1.15 | 1.15 | .57 * |
| *25-34 years (ref)* | 1 | 1 | 1 |  | 1 | 1 |
| *35-39 years* | .84 ** | .82 ** | .90 | .78 *** | .78 *** | .88 |
| *40-44 years* | .87 + | .82 * | 1.04 | .84 + | .83 * | 1.29 |
| *45-49 years* | .69 ** | .65 *** | .88 | .73 * | .72 * | 1.40 |
| *50-59 years* | .78 | .73 + | 1.13 | .70 + | .69 * | 1.36 |
| *> 60 years* | .70 | .64 |  | .70 | .66 |  |
| Child’s age at mum’s death |  |  |  |  |  |  |
| *< 1 year* | 1.02 | 1.09 | 1.79 | .80 | .86 | 1.79 |
| *1-4 years* | 1.30 * | 1.28 * | 1.05 | 1.11 | 1.12 | 1.10 |
| *5-14 years* | 1.08 | 1.09 | .82 | 1.03 | 1.03 | .83 |
| *> 15 years (ref)* | 1 | 1 | 1 |  | 1 | 1 |
| Child’s age at dad’s death |  |  |  |  |  |  |
| *< 1 year* | 1.45 | 1.46 | 1.18 | 1.44 | 1.41 | 1.21 |
| *1-4 years* | 1.20 | 1.20 | .85 | 1.28 + | 1.27 + | 1.35 |
| *5-14 years* | .99 | 1.00 | .85 | .95 | .96 | 1.10 |
| *> 15 years (ref)* | 1 | 1 | 1 |  | 1 | 1 |
| Model parameters |  |  |  |  |  |  |
| LR chi² | 88.90 | 160.50 | 90.55 | 136.97 | 154.08 | 88.58 |
| Chi² | 0.0000 | 0.0000 | 0.0000 | 0.0000 | 0.0000 | 0.0000 |
| Log likelihood | -13291.74 | -13255.944 | -1072.2759 | -11666.117 | -11657.559 | -873.46121 |

iwt = model specification if covariates show an interaction with time

Significance: *** p < 0.001; ** p < 0.01; * p < 0.05; + p < 0.1
